# Bis[4-(4-pyridyl)pyridinium] (4-carboxy­pyridine-2,6-dicarboxyl­ato-κ^3^ *O* ^2^,*N*,*O* ^6^)(pyridine-2,4,6-tricarboxyl­ato-κ^3^ *O* ^2^,*N*,*O* ^6^)ferrate(III) trihydrate

**DOI:** 10.1107/S1600536809010514

**Published:** 2009-03-28

**Authors:** Li Zhao, You-Ren Dong, Hong-Zhen Xie

**Affiliations:** a State Key Laboratory Base of Novel Functional Materials and Preparation Science, Faculty of Materials Science and Chemical Engineering, Institute of Solid Materials Chemistry, Ningbo University, Zhejiang 315211, People’s Republic of China

## Abstract

In the title salt, (C_10_H_9_N_2_)_2_[Fe(C_8_H_2_NO_6_)(C_8_H_3_NO_6_)]·3H_2_O, the Fe^III^ atom is *O*,*N*,*O*′-chelated by dianionic and trianionic ligands in a slightly distorted octa­hedral coordination geometry. The cations and ferrate anions are linked into a layered structure; the layers are connected through the uncoordinated water mol­ecules into a hydrogen-bonded three-dimensional supra­molecular structure. One of the uncoordinated water molecules is disordered around an inversion centre and was refined with half-occupancy for each position.

## Related literature

For the design and synthesis of coordination polymer complexes and their potential applications, see: Kaneko *et al.* (2007 ▶); Li *et al.* (2008 ▶); Lin *et al.* (2009 ▶). For the H_3_ptc ligand, see: Ghosh & Bharadwaj (2006 ▶); Lin *et al.* (2007 ▶); Syper *et al.* (1980 ▶).
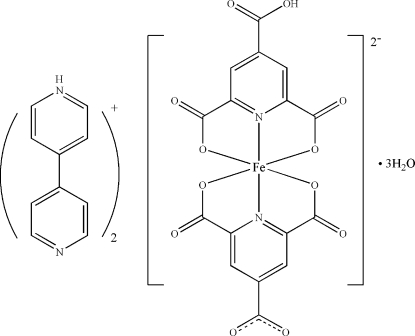


## Experimental

### 

#### Crystal data

(C_10_H_9_N_2_)_2_[Fe(C_8_H_2_NO_6_)(C_8_H_3_NO_6_)]·3H_2_O
*M* *_r_* = 841.50
Triclinic, 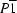

*a* = 10.568 (2) Å
*b* = 12.386 (3) Å
*c* = 14.344 (3) Å
α = 77.13 (3)°
β = 79.82 (3)°
γ = 76.15 (3)°
*V* = 1761.9 (6) Å^3^
*Z* = 2
Mo *K*α radiation
μ = 0.51 mm^−1^
*T* = 293 K
0.35 × 0.24 × 0.17 mm

#### Data collection

Rigaku R-AXIS RAPID diffractometer
Absorption correction: multi-scan (*ABSCOR*; Higashi, 1995 ▶) *T*_min_ = 0.860, *T*_max_ = 0.913
17019 measured reflections
7934 independent reflections
5458 reflections with *I* > 2σ(*I*)
*R*_int_ = 0.040

#### Refinement

*R*[*F* ^2^ > 2σ(*F* ^2^)] = 0.051
*wR*(*F* ^2^) = 0.138
*S* = 1.06
7934 reflections
526 parameters
H-atom parameters constrained
Δρ_max_ = 0.41 e Å^−3^
Δρ_min_ = −0.41 e Å^−3^

### 

Data collection: *RAPID-AUTO* (Rigaku, 1998 ▶); cell refinement: *RAPID-AUTO*; data reduction: *CrystalStructure* (Rigaku/MSC, 2004 ▶); program(s) used to solve structure: *SHELXS97* (Sheldrick, 2008 ▶); program(s) used to refine structure: *SHELXL97* (Sheldrick, 2008 ▶); molecular graphics: *SHELXL97*; software used to prepare material for publication: *SHELXTL* (Sheldrick, 2008 ▶).

## Supplementary Material

Crystal structure: contains datablocks global, I. DOI: 10.1107/S1600536809010514/ng2562sup1.cif

Structure factors: contains datablocks I. DOI: 10.1107/S1600536809010514/ng2562Isup2.hkl

Additional supplementary materials:  crystallographic information; 3D view; checkCIF report

## Figures and Tables

**Table 1:** Selected geometric parameters (Å, °)

Fe—O6	2.008 (2)
Fe—O7	2.012 (2)
Fe—O1	2.018 (2)
Fe—O12	2.026 (2)
Fe—N2	2.056 (2)
Fe—N1	2.058 (2)

**Table 2:** Hydrogen-bond geometry (Å, °)

*D*—H⋯*A*	*D*—H	H⋯*A*	*D*⋯*A*	*D*—H⋯*A*
O3—H3⋯O9^i^	0.82	1.64	2.454 (3)	172
N3—H3*A*⋯N5^ii^	0.86	1.93	2.741 (4)	158
N6—H6*A*⋯N4^iii^	0.86	1.84	2.694 (4)	170
O13*A*—H13*A*⋯O7^iv^	0.90	1.90	2.766 (4)	161
O13*B*—H13*B*⋯O4	0.78	2.17	2.821 (4)	142
O14—H14*A*⋯O11	0.85	2.16	2.973 (4)	160
O14—H14*B*⋯O5^v^	0.85	1.95	2.788 (4)	169
O15—H15*A*⋯O10	0.85	1.97	2.801 (3)	166
O15—H15*B*⋯O1^vi^	0.85	2.10	2.877 (3)	152

Symmetry codes: (i) 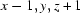; (ii) 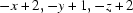; (iii) 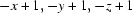; (iv) 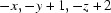; (v) 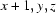; (vi) 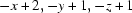.

**Fig. 1.**
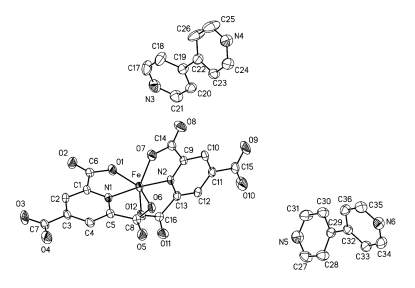
Aview of the complex molecule of the title compound, showing the atom-numbering scheme. Displacement ellipsoids are drawn at the 45% probability level. (Lattice water molecules and hydrogen atoms are all delected)

**Fig. 2.**
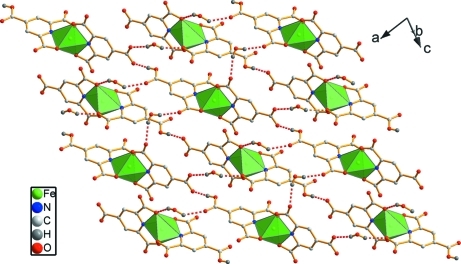
The two-dimensional layers constructed with [Fe(Hptc)(ptc)]2- by hydrogen bonds.

**Fig. 3.**
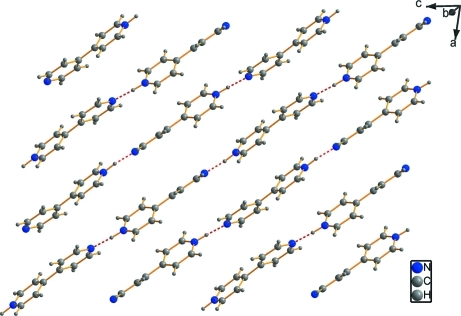
The two-dimensional layers constructed with Hbpy+ by hydrogen bonds and π-π stacking.

## supplementary crystallographic information

### Comment

Great interest has been focused on the rapidly expanding field of supramolecular chemistry and crystal engineering of the coordination polymers in reacent years because of their intriguing network topoloies as well as their potential application as functional materials in many areas such as separations and catalysis, gas storage, and magnetism (Kaneko *et al.*, 2007; Li *et al.*, 2008; Lin *et al.*, 2009). Pyridine-2,4,6-tricarboxylic acid (H_3_ptc) is a good building block for constructing supramolecular complex, which can link 3 d, 4f and 3 d-4f metal ions. However, plenty of researches have focused on the supramolecular chemistry and coordination polymers which only include single carboxylic acid ligands, whereas the studies and syntheses about the mixed-ligand compounds which contain two or two more ligands seem comparatively limited (Ghosh & Bharadwaj, 2006; Lin *et al.*, 2007). In this paper, we report the crystal structure of the title compound prepared from FeCl_2_.6H_2_O, H_3_ptc and 4,4'-bipyridine (4,4'-bpy).

The structure of title compound consists of [Fe(Hptc)(ptc)]^2-^ anions, Hbpy+ cations and lattice water molecules. In the anion, Hptc^2-^ and ptc^3-^ ligands are bound to one Fe(III) ion through pyridine N and deprotonated carboxylate O atoms at 2- and 6-positions, leading to a distorted octahedral geometry around the metal. The carboxylic groups at the 4-position of ptc ligands are uncoordinated. [Fe(Hptc)(ptc)]^2-^ anion is connected into two-dimensional layers through H-bonding interactions (Talbe 2). In the cationic part, the Hbpy^+^ ligands are not coordinated to metal ions. They are connected by N—H···N hydrogen-bonding and π-π stacking interactions to form another two-dimensional layers. The layers are futher linked into three-dimensional supramolecular structure by the intermolecular hydrogen bond interaction.

### Experimental

Pyridine-2,4,6-tricarboxylic acid (H_3_ptc) was synthesized by oxidization of pyridine-2,4,6-trimethyl with potassium permanganate according to a literature (Syper *et al.*, 1980).A mixture of H_3_ptc (0.110 g, 0.05 mmol), FeCl_2_.6H_2_O (0.126 g, 0.10 mmol), 4,4'-bpy (0.156 g, 0.10 mmol), 16 ml H_2_O and seven drops of triethylamine were loaded into a 23 ml Teflon-lined stainless autoclave, which was heated up to 120 °C, at which temperature the reactor was held for 3 days, and then cooled to room temperature. The reaction yielded brown block crystals of (I) in a yield of 10.32% based on FeCl_2_.6H_2_O. IR spectroscopic analysis (KBr, υ/cm^-1^): 3535(*m*), 3436(*m*), 1674(*s*), 1612(*m*), 1573(w), 1492(*m*), 1330(*s*), 1195(*s*), 1076(w), 1043(*m*), 1006(*m*), 931(w), 813(*s*), 779(w), 748(*m*), 771(w), 678(w).

### Refinement

H atoms bonded to C atoms were placed in geometrically calculated position and were refined using a riding model, with *U*_iso_(H) = 1.2 *U*_eq_(C). H atoms bonded to N and carboxyl O atoms were palced in geometrically calculated position and were refined using a riding model, with *U*_iso_(H) = 1.5 *U*_eq_(C). The water H atoms were found in a difference Fourier synthesis and were refined using a riding model, with the O—H distances fixed as 0.85±0.01 Å and with *U*_iso_(H) values set at 1.5 *U*eq(O). The O13 was disordered into two positions and treated as each occupation of 50%.

### Crystal data

**Table:**

(C_10_H_9_N_2_)_2_[Fe(C_8_H_2_NO_6_)(C_8_H_3_NO_6_)]·3H_2_O	*Z* = 2
*M**_r_* = 841.50	*F*(000) = 866
Triclinic, *P*1	*D*_x_ = 1.586 Mg m^−^^3^
Hall symbol: -P 1	Mo *K*α radiation, λ = 0.71073 Å
*a* = 10.568 (2) Å	Cell parameters from 17019 reflections
*b* = 12.386 (3) Å	θ = 3.2–27.5°
*c* = 14.344 (3) Å	µ = 0.51 mm^−^^1^
α = 77.13 (3)°	*T* = 293 K
β = 79.82 (3)°	Block, brown
γ = 76.15 (3)°	0.35 × 0.24 × 0.17 mm
*V* = 1761.9 (6) Å^3^	

### Data collection

**Table:**

Rigaku R-AXIS RAPID diffractometer	7934 independent reflections
Radiation source: fine-focus sealed tube	5458 reflections with *I* > 2σ(*I*)
graphite	*R*_int_ = 0.040
Detector resolution: 0 pixels mm^-1^	θ_max_ = 27.5°, θ_min_ = 3.1°
ω scans	*h* = −13→13
Absorption correction: multi-scan (*ABSCOR*; Higashi, 1995)	*k* = −15→16
*T*_min_ = 0.860, *T*_max_ = 0.913	*l* = −17→18
17019 measured reflections	

### Refinement

**Table:**

Refinement on *F*^2^	Primary atom site location: structure-invariant direct methods
Least-squares matrix: full	Secondary atom site location: difference Fourier map
*R*[*F*^2^ > 2σ(*F*^2^)] = 0.051	Hydrogen site location: inferred from neighbouring sites
*wR*(*F*^2^) = 0.138	H-atom parameters constrained
*S* = 1.06	*w* = 1/[σ^2^(*F*_o_^2^) + (0.0677*P*)^2^ + 0.2949*P*] where *P* = (*F*_o_^2^ + 2*F*_c_^2^)/3
7934 reflections	(Δ/σ)_max_ = 0.018
526 parameters	Δρ_max_ = 0.41 e Å^−^^3^
0 restraints	Δρ_min_ = −0.41 e Å^−^^3^

### Special details

**Table:**

Geometry. All e.s.d.'s (except the e.s.d. in the dihedral angle between two l.s. planes) are estimated using the full covariance matrix. The cell e.s.d.'s are taken into account individually in the estimation of e.s.d.'s in distances, angles and torsion angles; correlations between e.s.d.'s in cell parameters are only used when they are defined by crystal symmetry. An approximate (isotropic) treatment of cell e.s.d.'s is used for estimating e.s.d.'s involving l.s. planes.
Refinement. Refinement of *F*^2^ against ALL reflections. The weighted *R*-factor *wR* and goodness of fit *S* are based on *F*^2^, conventional *R*-factors *R* are based on *F*, with *F* set to zero for negative *F*^2^. The threshold expression of *F*^2^ > σ(*F*^2^) is used only for calculating *R*-factors(gt) *etc*. and is not relevant to the choice of reflections for refinement. *R*-factors based on *F*^2^ are statistically about twice as large as those based on *F*, and *R*- factors based on ALL data will be even larger.

### Fractional atomic coordinates and isotropic or equivalent isotropic displacement parameters (Å^2^)

**Table:**

	*x*	*y*	*z*	*U*_iso_*/*U*_eq_	Occ. (<1)
Fe	0.48489 (4)	0.50845 (4)	0.74947 (3)	0.02949 (13)	
N1	0.3459 (2)	0.51476 (19)	0.86945 (15)	0.0271 (5)	
N2	0.6260 (2)	0.52164 (19)	0.63117 (14)	0.0253 (5)	
C1	0.3767 (3)	0.4481 (2)	0.95274 (18)	0.0272 (6)	
C2	0.2896 (3)	0.4545 (2)	1.03624 (18)	0.0299 (6)	
H2	0.3096	0.4081	1.0945	0.036*	
C3	0.1708 (3)	0.5328 (2)	1.03055 (18)	0.0300 (6)	
C4	0.1416 (3)	0.6013 (2)	0.94256 (19)	0.0310 (6)	
H4	0.0624	0.6535	0.9381	0.037*	
C5	0.2338 (3)	0.5894 (2)	0.86208 (18)	0.0286 (6)	
C6	0.5121 (3)	0.3728 (2)	0.94093 (19)	0.0308 (6)	
O1	0.57272 (18)	0.39221 (17)	0.85399 (13)	0.0358 (5)	
O2	0.5550 (2)	0.30376 (19)	1.00811 (15)	0.0450 (5)	
C7	0.0695 (3)	0.5450 (3)	1.1186 (2)	0.0335 (6)	
O3	0.1080 (2)	0.4852 (2)	1.19749 (14)	0.0525 (6)	
H3	0.0457	0.4873	1.2407	0.079*	
O4	−0.0370 (2)	0.6083 (2)	1.10993 (15)	0.0529 (6)	
C8	0.2268 (3)	0.6545 (2)	0.75967 (19)	0.0311 (6)	
O5	0.1333 (2)	0.73086 (19)	0.73936 (14)	0.0436 (5)	
O6	0.32849 (19)	0.62091 (18)	0.70084 (12)	0.0352 (5)	
C9	0.6408 (2)	0.4532 (2)	0.56858 (18)	0.0266 (6)	
C10	0.7329 (3)	0.4614 (2)	0.48723 (18)	0.0294 (6)	
H10	0.7429	0.4149	0.4426	0.035*	
C11	0.8105 (3)	0.5417 (2)	0.47426 (18)	0.0299 (6)	
C12	0.7941 (3)	0.6101 (2)	0.54191 (18)	0.0301 (6)	
H12	0.8464	0.6629	0.5345	0.036*	
C13	0.6987 (2)	0.5981 (2)	0.62030 (18)	0.0271 (6)	
C14	0.5477 (3)	0.3726 (2)	0.60007 (19)	0.0317 (6)	
O7	0.47146 (19)	0.38846 (18)	0.67958 (14)	0.0379 (5)	
O8	0.5477 (2)	0.3013 (2)	0.55424 (17)	0.0524 (6)	
C15	0.9150 (3)	0.5546 (3)	0.38788 (19)	0.0356 (7)	
O9	0.9347 (2)	0.4803 (2)	0.33614 (16)	0.0558 (7)	
O10	0.9727 (2)	0.6326 (2)	0.37478 (17)	0.0595 (7)	
C16	0.6609 (3)	0.6649 (2)	0.70080 (19)	0.0305 (6)	
O11	0.7138 (2)	0.74272 (19)	0.69996 (15)	0.0441 (5)	
O12	0.56886 (19)	0.63358 (17)	0.76424 (13)	0.0360 (5)	
N3	0.9300 (3)	0.0772 (3)	0.6608 (2)	0.0529 (8)	
H3A	0.9740	0.0865	0.7025	0.079*	
C17	0.8781 (4)	−0.0123 (4)	0.6781 (2)	0.0579 (10)	
H17	0.8887	−0.0644	0.7354	0.069*	
C18	0.8083 (4)	−0.0307 (3)	0.6131 (2)	0.0556 (10)	
H18	0.7717	−0.0945	0.6263	0.067*	
C19	0.7928 (3)	0.0467 (3)	0.5278 (2)	0.0373 (7)	
C20	0.8491 (3)	0.1404 (3)	0.5113 (2)	0.0422 (7)	
H20	0.8415	0.1932	0.4542	0.051*	
C21	0.9160 (3)	0.1548 (3)	0.5797 (3)	0.0512 (9)	
H21	0.9519	0.2187	0.5697	0.061*	
N4	0.5820 (3)	−0.0089 (3)	0.3213 (2)	0.0502 (7)	
C22	0.7179 (3)	0.0271 (3)	0.4566 (2)	0.0380 (7)	
C23	0.6505 (3)	0.1171 (3)	0.3962 (2)	0.0430 (7)	
H23	0.6490	0.1913	0.4003	0.052*	
C24	0.5854 (3)	0.0946 (3)	0.3297 (2)	0.0476 (8)	
H24	0.5414	0.1555	0.2886	0.057*	
C25	0.6459 (5)	−0.0947 (4)	0.3793 (3)	0.0829 (15)	
H25	0.6458	−0.1680	0.3735	0.100*	
C26	0.7133 (5)	−0.0797 (3)	0.4485 (3)	0.0783 (15)	
H26	0.7552	−0.1423	0.4893	0.094*	
N5	0.9437 (3)	0.9490 (3)	1.1818 (2)	0.0495 (7)	
C27	0.8663 (3)	1.0491 (3)	1.1540 (2)	0.0491 (9)	
H27	0.8631	1.1076	1.1858	0.059*	
C28	0.7908 (3)	1.0700 (3)	1.0802 (2)	0.0443 (8)	
H28	0.7390	1.1414	1.0619	0.053*	
C29	0.7940 (3)	0.9821 (2)	1.0337 (2)	0.0320 (6)	
C30	0.8776 (3)	0.8807 (3)	1.0597 (2)	0.0418 (7)	
H30	0.8854	0.8217	1.0273	0.050*	
C31	0.9503 (3)	0.8665 (3)	1.1342 (3)	0.0509 (9)	
H31	1.0059	0.7969	1.1518	0.061*	
N6	0.5522 (3)	1.0162 (2)	0.81949 (19)	0.0445 (7)	
H6A	0.5044	1.0219	0.7753	0.067*	
C32	0.7079 (3)	0.9959 (3)	0.9582 (2)	0.0324 (6)	
C33	0.6801 (3)	1.0959 (3)	0.8931 (2)	0.0403 (7)	
H33	0.7144	1.1574	0.8958	0.048*	
C34	0.6010 (3)	1.1043 (3)	0.8238 (2)	0.0452 (8)	
H34	0.5817	1.1718	0.7799	0.054*	
C35	0.5763 (3)	0.9191 (3)	0.8826 (2)	0.0433 (8)	
H35	0.5400	0.8592	0.8786	0.052*	
C36	0.6533 (3)	0.9062 (3)	0.9529 (2)	0.0394 (7)	
H36	0.6690	0.8383	0.9967	0.047*	
O14	0.8797 (3)	0.7658 (3)	0.8387 (2)	0.0770 (9)	
H14A	0.8215	0.7521	0.8116	0.116*	
H14B	0.9524	0.7531	0.8024	0.116*	
O15	1.1872 (2)	0.7054 (2)	0.25415 (18)	0.0571 (7)	
H15A	1.1225	0.6765	0.2841	0.086*	
H15B	1.2405	0.6626	0.2191	0.086*	
O13A	−0.299 (4)	0.735 (3)	1.201 (2)	0.087 (5)	0.50
O13B	−0.293 (4)	0.719 (3)	1.174 (2)	0.087 (5)	0.50
H13A	−0.3480	0.6982	1.2502	0.131*	
H13B	−0.2432	0.6654	1.1609	0.131*	

### Atomic displacement parameters (Å^2^)

**Table:**

	*U*^11^	*U*^22^	*U*^33^	*U*^12^	*U*^13^	*U*^23^
Fe	0.0310 (2)	0.0340 (2)	0.0201 (2)	−0.00864 (17)	0.00912 (14)	−0.00606 (16)
N1	0.0288 (12)	0.0288 (12)	0.0202 (10)	−0.0050 (10)	0.0050 (8)	−0.0047 (9)
N2	0.0270 (11)	0.0293 (12)	0.0175 (10)	−0.0071 (9)	0.0045 (8)	−0.0043 (9)
C1	0.0317 (14)	0.0284 (15)	0.0196 (12)	−0.0071 (11)	0.0038 (10)	−0.0052 (11)
C2	0.0357 (15)	0.0335 (16)	0.0178 (12)	−0.0097 (12)	0.0040 (10)	−0.0026 (11)
C3	0.0340 (15)	0.0350 (16)	0.0213 (13)	−0.0107 (12)	0.0070 (10)	−0.0106 (12)
C4	0.0275 (14)	0.0339 (16)	0.0296 (14)	−0.0021 (12)	0.0021 (11)	−0.0111 (12)
C5	0.0312 (14)	0.0297 (15)	0.0228 (13)	−0.0055 (12)	0.0025 (10)	−0.0061 (11)
C6	0.0355 (15)	0.0304 (16)	0.0254 (13)	−0.0082 (12)	0.0027 (11)	−0.0066 (12)
O1	0.0309 (10)	0.0404 (12)	0.0280 (10)	−0.0012 (9)	0.0100 (8)	−0.0074 (9)
O2	0.0454 (13)	0.0424 (13)	0.0325 (11)	0.0073 (10)	0.0005 (9)	0.0016 (10)
C7	0.0355 (16)	0.0363 (17)	0.0270 (14)	−0.0091 (13)	0.0094 (11)	−0.0113 (12)
O3	0.0432 (13)	0.0707 (17)	0.0273 (11)	−0.0002 (12)	0.0159 (9)	−0.0045 (11)
O4	0.0406 (13)	0.0621 (16)	0.0411 (12)	0.0063 (12)	0.0118 (10)	−0.0107 (12)
C8	0.0367 (16)	0.0336 (16)	0.0223 (13)	−0.0077 (13)	−0.0017 (11)	−0.0050 (12)
O5	0.0447 (13)	0.0442 (14)	0.0337 (11)	0.0016 (11)	−0.0047 (9)	−0.0022 (10)
O6	0.0388 (11)	0.0427 (12)	0.0196 (9)	−0.0065 (9)	0.0032 (8)	−0.0046 (8)
C9	0.0289 (14)	0.0282 (15)	0.0208 (12)	−0.0066 (11)	0.0033 (10)	−0.0054 (11)
C10	0.0287 (14)	0.0369 (16)	0.0207 (12)	−0.0055 (12)	0.0031 (10)	−0.0078 (11)
C11	0.0278 (14)	0.0339 (16)	0.0225 (13)	−0.0061 (12)	0.0054 (10)	−0.0013 (11)
C12	0.0326 (15)	0.0305 (15)	0.0251 (13)	−0.0118 (12)	0.0032 (11)	−0.0009 (11)
C13	0.0278 (14)	0.0290 (15)	0.0219 (13)	−0.0071 (11)	0.0015 (10)	−0.0016 (11)
C14	0.0342 (15)	0.0342 (16)	0.0267 (14)	−0.0121 (13)	0.0047 (11)	−0.0070 (12)
O7	0.0385 (11)	0.0427 (13)	0.0330 (10)	−0.0194 (10)	0.0127 (8)	−0.0104 (9)
O8	0.0595 (15)	0.0541 (15)	0.0543 (14)	−0.0294 (12)	0.0149 (11)	−0.0308 (12)
C15	0.0340 (15)	0.0437 (18)	0.0253 (14)	−0.0104 (14)	0.0064 (11)	−0.0043 (13)
O9	0.0602 (15)	0.0702 (17)	0.0390 (13)	−0.0285 (13)	0.0272 (11)	−0.0246 (12)
O10	0.0616 (15)	0.0712 (18)	0.0495 (14)	−0.0384 (14)	0.0268 (12)	−0.0193 (13)
C16	0.0352 (15)	0.0272 (15)	0.0279 (14)	−0.0060 (12)	−0.0013 (11)	−0.0056 (12)
O11	0.0546 (14)	0.0407 (13)	0.0431 (12)	−0.0207 (11)	0.0018 (10)	−0.0153 (10)
O12	0.0451 (12)	0.0380 (12)	0.0260 (10)	−0.0130 (10)	0.0076 (8)	−0.0131 (9)
N3	0.0578 (18)	0.066 (2)	0.0462 (17)	−0.0192 (16)	−0.0176 (14)	−0.0182 (16)
C17	0.072 (3)	0.068 (3)	0.0364 (18)	−0.023 (2)	−0.0220 (17)	0.0058 (17)
C18	0.074 (3)	0.053 (2)	0.0473 (19)	−0.034 (2)	−0.0233 (17)	0.0104 (17)
C19	0.0395 (17)	0.0368 (18)	0.0388 (16)	−0.0089 (14)	−0.0146 (13)	−0.0047 (13)
C20	0.054 (2)	0.0351 (18)	0.0424 (17)	−0.0150 (15)	−0.0144 (14)	−0.0053 (14)
C21	0.055 (2)	0.053 (2)	0.056 (2)	−0.0212 (18)	−0.0125 (16)	−0.0168 (18)
N4	0.0571 (18)	0.0484 (18)	0.0533 (17)	−0.0110 (14)	−0.0291 (14)	−0.0092 (14)
C22	0.0448 (17)	0.0364 (18)	0.0375 (16)	−0.0154 (14)	−0.0135 (13)	−0.0028 (13)
C23	0.055 (2)	0.0358 (18)	0.0409 (17)	−0.0031 (15)	−0.0181 (14)	−0.0100 (14)
C24	0.049 (2)	0.044 (2)	0.0485 (19)	0.0038 (16)	−0.0258 (15)	−0.0067 (16)
C25	0.127 (4)	0.038 (2)	0.106 (4)	−0.024 (2)	−0.078 (3)	−0.004 (2)
C26	0.121 (4)	0.032 (2)	0.100 (3)	−0.018 (2)	−0.085 (3)	0.007 (2)
N5	0.0512 (17)	0.056 (2)	0.0476 (16)	−0.0099 (15)	−0.0234 (13)	−0.0100 (14)
C27	0.053 (2)	0.052 (2)	0.050 (2)	−0.0059 (17)	−0.0173 (16)	−0.0223 (17)
C28	0.0459 (18)	0.0380 (19)	0.053 (2)	−0.0032 (15)	−0.0188 (15)	−0.0139 (16)
C29	0.0312 (15)	0.0330 (16)	0.0317 (14)	−0.0065 (12)	−0.0058 (11)	−0.0047 (12)
C30	0.0453 (18)	0.0384 (18)	0.0449 (18)	−0.0040 (14)	−0.0157 (14)	−0.0119 (15)
C31	0.054 (2)	0.041 (2)	0.061 (2)	−0.0005 (16)	−0.0284 (17)	−0.0111 (17)
N6	0.0439 (15)	0.0547 (18)	0.0402 (14)	−0.0101 (13)	−0.0208 (12)	−0.0078 (13)
C32	0.0330 (15)	0.0345 (17)	0.0311 (14)	−0.0061 (12)	−0.0064 (11)	−0.0082 (12)
C33	0.0451 (18)	0.0387 (18)	0.0398 (17)	−0.0109 (14)	−0.0113 (13)	−0.0063 (14)
C34	0.051 (2)	0.044 (2)	0.0418 (18)	−0.0082 (16)	−0.0191 (14)	−0.0015 (15)
C35	0.0421 (18)	0.046 (2)	0.0494 (19)	−0.0134 (15)	−0.0148 (14)	−0.0123 (16)
C36	0.0435 (18)	0.0368 (18)	0.0397 (16)	−0.0118 (14)	−0.0124 (13)	−0.0018 (14)
O14	0.0659 (18)	0.091 (2)	0.0760 (19)	−0.0138 (17)	−0.0019 (15)	−0.0274 (18)
O15	0.0461 (14)	0.0489 (15)	0.0652 (15)	−0.0081 (12)	0.0163 (11)	−0.0084 (12)
O13A	0.075 (4)	0.063 (8)	0.101 (15)	−0.016 (5)	0.050 (8)	−0.016 (6)
O13B	0.075 (4)	0.063 (8)	0.101 (15)	−0.016 (5)	0.050 (8)	−0.016 (6)

### Geometric parameters (Å, °)

**Table:**

Fe—O6	2.008 (2)	C18—H18	0.9300
Fe—O7	2.012 (2)	C19—C20	1.384 (4)
Fe—O1	2.018 (2)	C19—C22	1.488 (4)
Fe—O12	2.026 (2)	C20—C21	1.371 (4)
Fe—N2	2.056 (2)	C20—H20	0.9300
Fe—N1	2.058 (2)	C21—H21	0.9300
N1—C5	1.321 (3)	N4—C25	1.318 (5)
N1—C1	1.336 (3)	N4—C24	1.323 (4)
N2—C13	1.324 (3)	C22—C26	1.367 (5)
N2—C9	1.332 (3)	C22—C23	1.381 (4)
C1—C2	1.380 (3)	C23—C24	1.380 (4)
C1—C6	1.514 (4)	C23—H23	0.9300
C2—C3	1.393 (4)	C24—H24	0.9300
C2—H2	0.9300	C25—C26	1.386 (5)
C3—C4	1.394 (4)	C25—H25	0.9300
C3—C7	1.515 (3)	C26—H26	0.9300
C4—C5	1.383 (3)	N5—C27	1.332 (4)
C4—H4	0.9300	N5—C31	1.332 (4)
C5—C8	1.515 (4)	C27—C28	1.379 (4)
C6—O2	1.212 (3)	C27—H27	0.9300
C6—O1	1.296 (3)	C28—C29	1.389 (4)
C7—O4	1.216 (4)	C28—H28	0.9300
C7—O3	1.281 (4)	C29—C30	1.369 (4)
O3—H3	0.8200	C29—C32	1.491 (4)
C8—O5	1.220 (3)	C30—C31	1.380 (4)
C8—O6	1.289 (3)	C30—H30	0.9300
C9—C10	1.383 (3)	C31—H31	0.9300
C9—C14	1.510 (4)	N6—C34	1.331 (4)
C10—C11	1.397 (4)	N6—C35	1.334 (4)
C10—H10	0.9300	N6—H6A	0.8600
C11—C12	1.388 (4)	C32—C33	1.379 (4)
C11—C15	1.514 (3)	C32—C36	1.390 (4)
C12—C13	1.378 (3)	C33—C34	1.379 (4)
C12—H12	0.9300	C33—H33	0.9300
C13—C16	1.510 (4)	C34—H34	0.9300
C14—O8	1.213 (3)	C35—C36	1.364 (4)
C14—O7	1.299 (3)	C35—H35	0.9300
C15—O10	1.225 (4)	C36—H36	0.9300
C15—O9	1.265 (4)	O14—H14A	0.8499
C16—O11	1.221 (3)	O14—H14B	0.8508
C16—O12	1.279 (3)	O15—H15A	0.8517
N3—C17	1.309 (5)	O15—H15B	0.8520
N3—C21	1.339 (5)	O13A—H13A	0.9035
N3—H3A	0.8600	O13A—H13B	1.1256
C17—C18	1.372 (5)	O13B—H13A	1.1455
C17—H17	0.9300	O13B—H13B	0.7764
C18—C19	1.383 (4)		
			
O6—Fe—O7	95.18 (9)	C16—O12—Fe	120.48 (17)
O6—Fe—O1	151.77 (7)	C17—N3—C21	121.4 (3)
O7—Fe—O1	92.17 (9)	C17—N3—H3A	119.3
O6—Fe—O12	91.68 (9)	C21—N3—H3A	119.3
O7—Fe—O12	151.67 (8)	N3—C17—C18	121.1 (3)
O1—Fe—O12	94.66 (9)	N3—C17—H17	119.5
O6—Fe—N2	102.03 (9)	C18—C17—H17	119.5
O7—Fe—N2	75.81 (8)	C17—C18—C19	119.5 (3)
O1—Fe—N2	106.20 (8)	C17—C18—H18	120.3
O12—Fe—N2	75.88 (8)	C19—C18—H18	120.3
O6—Fe—N1	75.78 (9)	C18—C19—C20	118.3 (3)
O7—Fe—N1	110.98 (8)	C18—C19—C22	119.9 (3)
O1—Fe—N1	76.14 (8)	C20—C19—C22	121.9 (3)
O12—Fe—N1	97.35 (8)	C21—C20—C19	119.5 (3)
N2—Fe—N1	172.91 (9)	C21—C20—H20	120.2
C5—N1—C1	122.9 (2)	C19—C20—H20	120.2
C5—N1—Fe	118.62 (18)	N3—C21—C20	120.3 (3)
C1—N1—Fe	118.31 (18)	N3—C21—H21	119.9
C13—N2—C9	122.6 (2)	C20—C21—H21	119.9
C13—N2—Fe	118.47 (16)	C25—N4—C24	117.7 (3)
C9—N2—Fe	118.94 (17)	C26—C22—C23	117.7 (3)
N1—C1—C2	120.3 (2)	C26—C22—C19	121.5 (3)
N1—C1—C6	111.8 (2)	C23—C22—C19	120.8 (3)
C2—C1—C6	127.9 (3)	C24—C23—C22	118.6 (3)
C1—C2—C3	118.0 (2)	C24—C23—H23	120.7
C1—C2—H2	121.0	C22—C23—H23	120.7
C3—C2—H2	121.0	N4—C24—C23	123.6 (3)
C2—C3—C4	120.4 (2)	N4—C24—H24	118.2
C2—C3—C7	121.4 (3)	C23—C24—H24	118.2
C4—C3—C7	118.2 (3)	N4—C25—C26	122.5 (4)
C5—C4—C3	118.1 (3)	N4—C25—H25	118.8
C5—C4—H4	120.9	C26—C25—H25	118.8
C3—C4—H4	120.9	C22—C26—C25	119.9 (4)
N1—C5—C4	120.3 (2)	C22—C26—H26	120.1
N1—C5—C8	111.4 (2)	C25—C26—H26	120.1
C4—C5—C8	128.3 (3)	C27—N5—C31	118.0 (3)
O2—C6—O1	126.0 (3)	N5—C27—C28	123.1 (3)
O2—C6—C1	121.2 (2)	N5—C27—H27	118.4
O1—C6—C1	112.7 (2)	C28—C27—H27	118.4
C6—O1—Fe	120.88 (18)	C27—C28—C29	118.4 (3)
O4—C7—O3	126.2 (2)	C27—C28—H28	120.8
O4—C7—C3	119.8 (3)	C29—C28—H28	120.8
O3—C7—C3	114.1 (3)	C30—C29—C28	118.4 (3)
C7—O3—H3	109.5	C30—C29—C32	120.1 (3)
O5—C8—O6	126.1 (3)	C28—C29—C32	121.5 (3)
O5—C8—C5	121.1 (2)	C29—C30—C31	119.6 (3)
O6—C8—C5	112.8 (2)	C29—C30—H30	120.2
C8—O6—Fe	120.84 (18)	C31—C30—H30	120.2
N2—C9—C10	120.4 (2)	N5—C31—C30	122.4 (3)
N2—C9—C14	111.4 (2)	N5—C31—H31	118.8
C10—C9—C14	128.2 (2)	C30—C31—H31	118.8
C9—C10—C11	117.9 (2)	C34—N6—C35	121.2 (3)
C9—C10—H10	121.0	C34—N6—H6A	119.4
C11—C10—H10	121.0	C35—N6—H6A	119.4
C12—C11—C10	120.1 (2)	C33—C32—C36	118.6 (3)
C12—C11—C15	118.8 (2)	C33—C32—C29	121.7 (3)
C10—C11—C15	121.1 (2)	C36—C32—C29	119.7 (3)
C13—C12—C11	118.6 (2)	C34—C33—C32	119.7 (3)
C13—C12—H12	120.7	C34—C33—H33	120.2
C11—C12—H12	120.7	C32—C33—H33	120.2
N2—C13—C12	120.4 (2)	N6—C34—C33	120.2 (3)
N2—C13—C16	111.8 (2)	N6—C34—H34	119.9
C12—C13—C16	127.8 (2)	C33—C34—H34	119.9
O8—C14—O7	125.4 (3)	N6—C35—C36	121.1 (3)
O8—C14—C9	121.8 (2)	N6—C35—H35	119.4
O7—C14—C9	112.8 (2)	C36—C35—H35	119.4
C14—O7—Fe	121.07 (17)	C35—C36—C32	119.2 (3)
O10—C15—O9	126.3 (3)	C35—C36—H36	120.4
O10—C15—C11	118.9 (3)	C32—C36—H36	120.4
O9—C15—C11	114.8 (3)	H14A—O14—H14B	107.2
O11—C16—O12	126.0 (2)	H15A—O15—H15B	112.8
O11—C16—C13	120.6 (2)	H13A—O13A—H13B	103.2
O12—C16—C13	113.4 (2)	H13A—O13B—H13B	110.8

### Hydrogen-bond geometry (Å, °)

**Table:**

*D*—H···*A*	*D*—H	H···*A*	*D*···*A*	*D*—H···*A*
O3—H3···O9^i^	0.82	1.64	2.454 (3)	172
N3—H3A···N5^ii^	0.86	1.93	2.741 (4)	158
N6—H6A···N4^iii^	0.86	1.84	2.694 (4)	170
O13A—H13A···O7^iv^	0.90	1.90	2.766 (4)	161
O13B—H13B···O4	0.78	2.17	2.821 (4)	142
O14—H14A···O11	0.85	2.16	2.973 (4)	160
O14—H14B···O5^v^	0.85	1.95	2.788 (4)	169
O15—H15A···O10	0.85	1.97	2.801 (3)	166
O15—H15B···O1^vi^	0.85	2.10	2.877 (3)	152

Symmetry codes: (i) *x*−1, *y*, *z*+1; (ii) −*x*+2, −*y*+1, −*z*+2; (iii) −*x*+1, −*y*+1, −*z*+1; (iv) −*x*, −*y*+1, −*z*+2;  (v) *x*+1, *y*, *z*; (vi) −*x*+2, −*y*+1, −*z*+1.
